# A Novel Calcium-Mediated EMT Pathway Controlled by Lipids: An Opportunity for Prostate Cancer Adjuvant Therapy

**DOI:** 10.3390/cancers11111814

**Published:** 2019-11-18

**Authors:** Sandy Figiel, Fanny Bery, Aurélie Chantôme, Delphine Fontaine, Côme Pasqualin, Véronique Maupoil, Isabelle Domingo, Roseline Guibon, Franck Bruyère, Marie Potier-Cartereau, Christophe Vandier, Gaëlle Fromont, Karine Mahéo

**Affiliations:** 1 Inserm N2C UMR1069, Université de Tours, F-37032 Tours cedex 1, France; sandy.figiel@gmail.com (S.F.); fannybery@hotmail.fr (F.B.); aurelie.chantome@med.univ-tours.fr (A.C.); delphine.fontaine@etu.univ-tours.fr (D.F.); isabelle.domingo@univ-tours.fr (I.D.); marie.potier-cartereau@univ-tours.fr (M.P.-C.); christophe.vandier@univ-tours.fr (C.V.); gaelle.fromont-hankard@univ-tours.fr (G.F.); 2 Faculté de Pharmacie, Université de Tours, F-37200 Tours, France; come.pasqualin@univ-tours.fr (C.P.); veronique.maupoil@univ-tours.fr (V.M.); 3 ERL CNRS 7003-EA 7349-Signalisation et transports ioniques membranaires (STIM), 37200 Tours, France; 4 Departments of Pathology and Urology, CHRU Bretonneau, F-37044 Tours cedex 9, France; roseline.guibon@univ-tours.fr (R.G.); f.bruyere@chu-tours.fr (F.B.)

**Keywords:** prostate cancer, linoleic acid, eicosapentaenoic acid, Zeb1, calcium

## Abstract

The composition of periprostatic adipose tissue (PPAT) has been shown to play a role in prostate cancer (PCa) progression. We recently reported an inverse association between PCa aggressiveness and elevated PPAT linoleic acid (LA) and eicosapentaenoic acid (EPA) content. In the present study, we identified a new signaling pathway with a positive feedback loop between the epithelial-to-mesenchymal transition (EMT) transcription factor Zeb1 and the Ca^2+^-activated K^+^ channel SK3, which leads to an amplification of Ca^2+^ entry and cellular migration. Using in vitro experiments and ex vivo cultures of human PCa slices, we demonstrated that LA and EPA exert anticancer effects, by modulating Ca^2+^ entry, which was involved in Zeb1 regulation and cancer cellular migration. This functional approach using human prostate tumors highlights the clinical relevance of our observations, and may allow us to consider the possibility of targeting cancer spread by altering the lipid microenvironment.

## 1. Introduction

Prostate cancer (PCa), which is the most commonly diagnosed non cutaneous cancer in men, is a highly heterogeneous disease with variable clinical outcomes [1]. The prostate is surrounded by a rim of periprostatic adipose tissue (PPAT) that may be infiltrated by cancer cells at the locally advanced disease stage (pT3) stage. Previous studies have supported an important role for PPAT in the modulation of PCa progression, which may be mainly mediated through adipokine release [2,3]. However, others adipocyte mediators, such as fatty acids (FA), are also likely to interact with cancer cells.

We recently demonstrated an association between aggressive PCa and the FA PPAT composition. A specific FA profile was associated with PCa aggressiveness, characterized by low levels of both the essential n-6 polyunsaturated FA (PUFA) linoleic acid (LA) and the n-3 PUFA eicosapentaenoic acid (EPA). Moreover, in vitro migration potential of PCa cell lines supplemented with FA extracts obtained from PPAT was inversely correlated with adipose tissue LA content [4]. Taken together, these data suggest a causal relationship between PPAT FA composition and PCa aggressiveness, but the mechanisms by which LA and EPA can impact cancer cell migration remain to be investigated.

Cancer progression is dependent on the epithelial-to-mesenchymal transition (EMT) process, which allows the de-differentiation of epithelial cells to a mesenchymal-like phenotype and increases cancer cell invasion and migration. Zeb1 (zinc finger enhancer binding protein) is a key EMT driver that can be induced not only by microenvironmental factors, including TGFβ and hypoxia, but also by other EMT transcription factors such as Snail [5]. Zeb1 has been shown to induce EMT in PCa cell lines and promote cancer cell migration and invasion [6]. Importantly, we previously demonstrated that Zeb1 expression levels in human PCa increased with disease progression from localized (pT2) to locally advanced (pT3) tumors and metastases. Moreover, positive Zeb1 staining in metastatic tissues was significantly associated with decreased overall survival [7].

Calcium (Ca^2+^) signaling plays a major role in several events driving cancer progression mainly by regulating cytosolic Ca^2+^ concentrations involved in cell migration [8]. Enhancement of Ca^2+^ entry has been associated with EMT in breast cancer cells [9]. Moreover, several Ca^2+^ channels have been shown to induce the expression of EMT markers [9] and are required for TGFβ-induced EMT [10]. In the PCa cell lines DU145 and PC3, the involvement of several Ca^2+^ channels has already been studied in different processes of tumor development and progression, including proliferation, differentiation, apoptosis and migration [11]. In non-excitable cells, Ca^2+^ entry from extracellular medium is mainly supported by a capacitive Ca^2+^ entry mechanism, also known as store-operated Ca^2+^ entry (SOCE), which is mediated by store-operated Ca^2+^ channels [12]. We have previously demonstrated a pivotal role for the SK3 channel, which is a Ca^2+^ activated K^+^ channel regulating Ca^2+^ entry and cancer cell migration [13]. SK3 has been shown to form complexes with Ca^2+^ channels, thereby leading to increased cytosolic Ca^2+^ concentrations in cancer cells and promoting metastases development [14]. Interestingly, SK3 is located in cholesterol-enriched membrane nanodomains (also known as lipid rafts), and its function is dependent on the lipid microenvironment [14].

The effects of PUFAs on intracellular Ca^2+^ concentration alterations mediated by ion channels have been studied in several pathologies, such as cardiovascular diseases and neurological disorders. For example, n-3 PUFAs have been shown to prevent arrhythmias by modulation of L-type Ca^2+^ channels and sodium–calcium exchanger activity [15,16]. These effects are likely to depend on the type of channel involved, the site of FAs interaction within the channel, and the type of PUFAs. Although in TRPV4 channels the amplitude of the current has been shown to increase following application of n-3 PUFAs, the stimulation of TRPM8 by cold or menthol is almost completely inhibited by the n-6 PUFA arachidonic acid [17].

In this study, we have investigated whether LA and EPA could impact PCa progression by modulating Ca^2+^ signaling and the EMT process. For this study, we used cell lines and human PCa slices, which is an adequate model that maintains both cancer cell heterogeneity and an intact microenvironment.

## 2. Results 

### 2.1. Linoleic Acid and Eicosapentaenoic Acid Inhibit Cell Migration Induced by TGFβ and the Expression of Zeb1 and Its Target Genes

**Figure 1:**
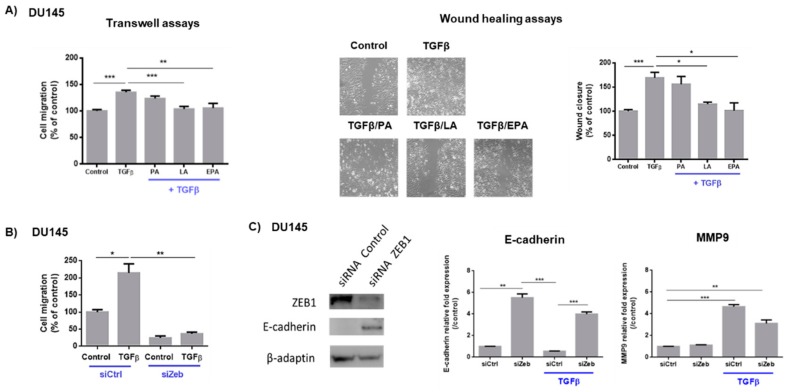
LA and EPA inhibit TGFβ-induced-migration, which is dependent of Zeb1. (**A**) LA and EPA inhibit TGFβ-induced cellular migration. DU145 cells were treated for 48 h with FA (LA, EPA, AP) (20 µM) and with TGFβ (10 ng/mL) and then used for transwell and wound healing migration assays performed for 24 h (in the presence of TGFβ and/or FA) (*N* = 3; *n* = 2). Results are expressed as mean ± SEM. Statistical differences are indicated: * *p* < 0.05; ** *p* < 0.01; *** *p* < 0.001 (Kruskal–Wallis; post-test: Dunn’s test). The scale of the photos is ×200 magnification. (**B**) Zeb1 is required for promigratory effect of TGFβ. siRNA-transfected cells (siCtrl, siZeb1) were treated for 48 h with TGFβ (10 ng/mL) and then used for transwell migration assay performed for 24 h (in the presence of TGFβ) (*N* = 3; *n* = 2). * *p* < 0.05; ** *p* < 0.01 (Kruskal–Wallis; post-test: Dunn’s test) (**C**) Effects of inhibition of Zeb1 expression on epithelial-to-mesenchymal transition (EMT) markers. siRNA-transfected DU145 cells (siCtrl, siZeb1) were treated or not for 48 h with TGFβ (10 ng/mL). qPCR results (mean ± SEM) are expressed in 2^-ΔΔCt^. (*N* = 3; *n* = 3). Statistical differences are indicated: ** *p* < 0.01; *** *p* < 0.001 (Kruskal–Wallis; post-test: Dunn’s test).

We first investigated whether the FA identified in our previous study to be inversely associated with PCa aggressiveness can directly regulate cell migration induced by TGFβ. Figure 1A shows that LA and EPA can abrogate TGFβ promigratory effect, as observed using two different cellular migration assays, whereas PA supplementation had no effect. In addition, FA had no effect on basal cellular migration in the absence of TGFβ treatment (Figure S1). We then investigated whether PCa cell migration induced by TGFβ is dependent on Zeb1. Suppression of Zeb1 strongly reduced the promigratory effect of TGFβ, indicating that Zeb1 is a key regulator of PCa cell migration (Figure 1B). Western blot analysis shows the protein expression level of Zeb1 and E-cadherin (an epithelial target gene of Zeb1) in siZeb1-transfected DU145 cells. The strong decrease of Zeb1 was associated with an increase in E-cadherin protein level. Zeb1 suppression also increased basal and TGFβ-induced E-cadherin mRNA level (Figure 1C). By contrast, Zeb1 suppression tended to decrease TGFβ-induced MMP9 expression (a mesenchymal target gene of Zeb1).

**Figure 2:**
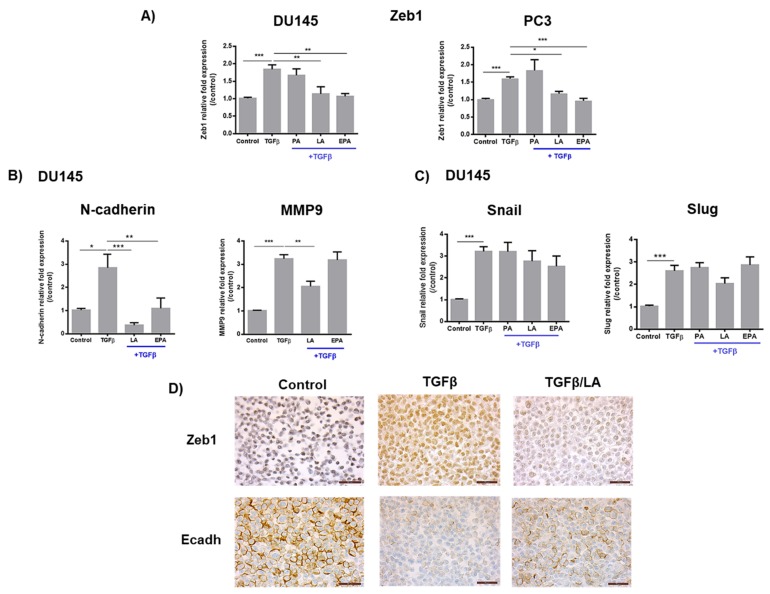
LA and EPA inhibit the TGFβ-induced Zeb1 and its target genes expression. (**A**–**C**) Zeb1, N-cadherin, MMP9, Snail, and Slug mRNA levels in the prostate cancer (PCa) cell line. Cells were treated for 48 h by TGFβ (10 ng/mL) ± FA (LA, EPA, AP) (20 µM). qPCR results (mean ± SEM) are expressed in 2^-ΔΔCt^. (*N* = 3; *n* = 3). Statistical differences are indicated: * *p* < 0.05; ** *p* < 0.01; *** *p* < 0.001 (Kruskal–Wallis; post-test: Dunn’s test). (**D**) Zeb1 and Ecadherin protein expression in DU145 PCa cells. Treatment with TGFβ (10 ng/mL) increased Zeb1 expression (from 30% to 100% positive cells) and decreased Ecadherin staining (from 90% to 25% positive cells). Addition of LA (60 µM) for 48 h led to decrease Zeb1 (40%) and to increase Ecadherin expression (70%), compared to TGFβ treatment alone (*N* = 3). Scale bars = 50 µm.

As observed in Figure 2A, TGFβ treatment increased the expression of Zeb1 by 1.7-fold. This effect was abrogated in LA and EPA-supplemented cells, but not after supplementation by PA. In addition, LA inhibited TGFβ-induced N-cadherin and MMP9 expression, whereas EPA treatment affected only N-cadherin mRNA levels (Figure 2B). By contrast, FA had no effect on the expression of other EMT transcription factors such as Snail and Slug (Figure 2C). All FA tested had no effect on basal Zeb1 expression (without TGFβ treatment) (Figure S2). Figure 2D shows representative images of Zeb1 and E-Cadherin protein expression by immunohistochemistry in the DU145 cells. LA supplementation strongly decreased TGFβ-induced Zeb1 staining in cancer cells. The decrease in E-cadherin expression induced by TGFβ was clearly reversed by LA.

### 2.2. LA and EPA Inhibit SK3 Expression Induced by TGFβ, and SK3 is Dependent on Zeb1 Expression

**Figure 3:**
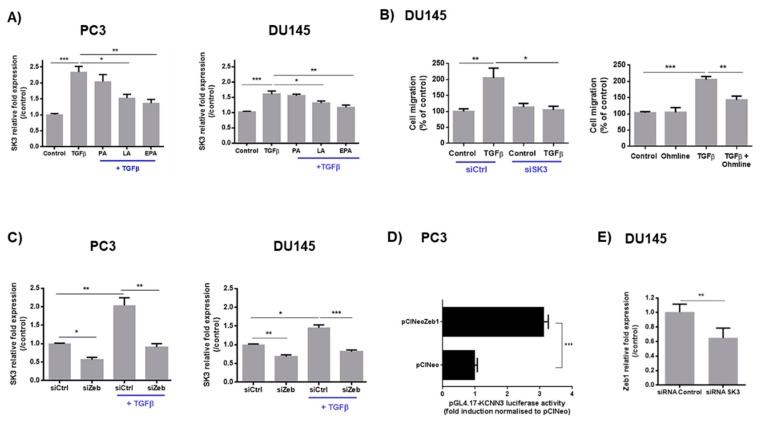
LA and EPA inhibit SK3 expression induced by TGFβ, and SK3 is dependent on Zeb1 expression. (**A**) LA and EPA inhibit TGFβ-induced SK3 mRNA level in PCa cells. Cells were treated for 48 h by TGFβ (10 ng/mL) ± FA (LA, EPA, AP) (20 µM). (*N* = 3; *n* = 3). * *p* < 0.05; ** *p* < 0.01; *** *p* < 0.001 (Kruskal–Wallis; post-test: Dunn’s test) (**B**) SK3 is required for promigratory effect of TGFβ. siRNA-transfected (siCtrl, siSK3) and cells treated with TGFβ (10 ng/mL) ± Ohmline (1 µM) for 48 h were used for transwell migration assay performed for 24 h (in the presence of TGFβ) (*N* = 3; *n* = 2). * *p* < 0.05; ** *p* < 0.01; *** *p* < 0.001 (Kruskal–Wallis; post-test: Dunn’s test) (**C**) Zeb1 regulates SK3 channel expression. SK3 mRNA levels in siCtrl and siZeb1-transfected cells and treated by TGFβ (10 ng/mL) for 48 h (qPCR analysis were performed 48 h post-transfection). Results are expressed as mean ± SEM. (*N* = 3; *n* = 3). Statistical differences are indicated: * *p* < 0.05; ** *p* < 0.01; *** *p* < 0.001 (Kruskal–Wallis; post-test: Dunn’s test). (**D**) Overexpression of Zeb1 enhanced *KCNN3* gene transcription. PC3 cells were cotransfected with pGL4.17-KCNN3, pRL-TK, and pCIneo (control condition) or pCIneoZeb1. Dual-luciferase reporter assays were performed 72 h after transfection. Results are normalized to control conditions and expressed as mean ± SEM. Statistical differences are indicated: *** *p* < 0.001 (t-test one way ANOVA). (*N* = 3). (**E**) SK3 regulates Zeb1 channel expression. Zeb1 mRNA levels in siCtrl and siSK3- transfected-cells (qPCR analysis were performed 48 h post-transfection). Results are expressed as mean ± SEM. (*N* = 3; *n* = 3). Statistical differences are indicated: ** *p* < 0.01 (Kruskal–Wallis; post-test: Dunn’s test).

We investigated whether SK3 channel could also be regulated by TGFβ and FA in PCa cell lines. As observed in Figure 3A, TGFβ increased the expression of the SK3 channel by ~2-fold. This effect was strongly reduced after incubation with LA and EPA. In contrast, FA supplementation had no effect on the expression of Ca^2+^ channels TRPC1, STIM1, Orai1, and Orai3 induced by TGFβ (Figure S3). No effect on SK3 basal expression was observed in the presence of FA (Figure S4).

We then investigated whether PCa cell migration induced by TGFβ is dependent on SK3. As observed in Figure 3B, suppression of SK3 strongly reduced the promigratory effect of TGFβ, indicating that SK3 is also a regulator of PCa cell migration. Similar results were obtained with a specific pharmacological inhibitor of SK3 (Ohmline, [14]).

To analyze the relationship between Zeb1 and SK3, we first used siZeb1-transfected cells. We observed that the suppression of Zeb1 expression reduced SK3 levels, and that TGFβ failed to induce SK3 expression in siZeb1-transfected cells. These data suggest that the induction of SK3 expression by TGFβ requires the presence of Zeb1 (Figure 3C). To determine whether SK3 is a target gene of Zeb1, we monitored gene transcription from a *KCNN3* (encoding SK3 channel) promoter–luciferase reporter constructs in PC3-transfected with the pCIneo-Zeb1 plasmid, which allows constitutive transcription of *Zeb1*. As predicted, overexpression of Zeb1 increased *KCNN3* promoter activity (by 3-fold) (Figure 3D). These results showed for the first time that the transcription factor Zeb1 promotes the expression of the SK3 channel. Interestingly, SK3 extinction decreased Zeb1 expression, suggesting a positive feedback loop (Figure 3E). 

### 2.3. Ca^2+^ Entry Is Required for the Upregulation of Zeb1 Expression and Is Inhibited by LA and EPA In Vitro and Ex Vivo

**Figure 4:**
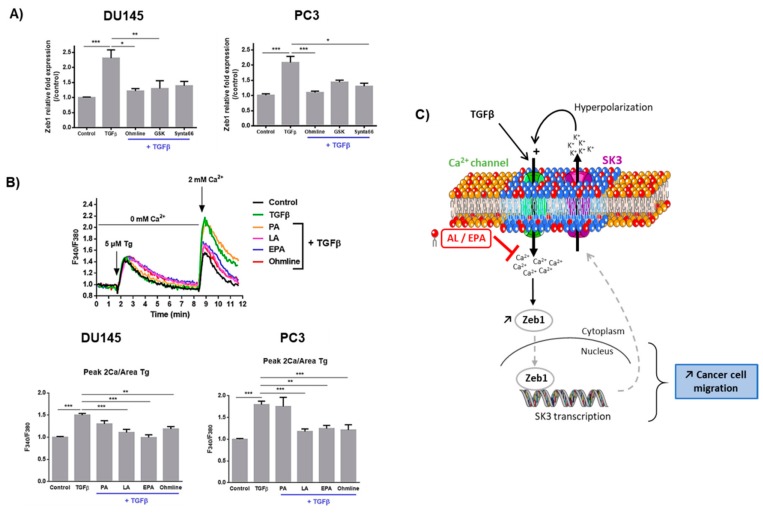
Ca^2+^ entry is required for TGFβ-induced Zeb1 expression, and TGFβ-induced SOCE is inhibited by LA and EPA. (**A**) Zeb1 expression is inhibited by an SK3 channel inhibitor and Ca^2+^ channel inhibitors. PCa cells were treated with TGFβ (10 ng/mL) ± GSK7975A (GSK in the figure), Synta66, or Ohmline (1 µM) for 48 h. Results are expressed as mean ± SEM. Statistical differences are indicated: * *p* < 0.05; ** *p* < 0.01; *** *p* < 0.001 (Kruskal–Wallis; post-test: Dunn). (*N* = 3; *n* = 3). (**B**) TGFβ-induced SOCE is inhibited by LA, EPA, and Ohmline. Fluorescence measurements and relative fluorescence of Ca^2+^ entry after intracellular Ca^2+^ store depletion by thapsigargin (Tg) in PCa cells pretreated for 48 h by Ohmline (1 µM), TGFβ (10 ng/mL), FA (20 µM). Histograms showing relative fluorescence of Ca^2+^ variations. (*N* = 3; *n* = 3). Statistical differences are indicated: ** *p* < 0, 01; *** *p* < 0,001 (Kruskal–Wallis; post-test: Dunn’s test). Results are expressed as mean ± SEM. (**C**) Proposed model for a positive feedback loop leading to PCa cellular migration, and inhibited by LA and EPA: (1) TGFβ increases calcium entry in PCa cells. (2) This calcium influx promotes expression of the transcription factor Zeb1 that targets the SK3 channel gene. (3) At the plasma membrane, the SK3 channel allows an increase in calcium entry by hyperpolarization of the plasma membrane. By incorporating into the membrane, LA and EPA inhibit this signaling pathway induced by TGFβ. These FA inhibit calcium entry, Zeb1, and SK3 expression and PCa cell migration.

Several studies have shown that EMT inducers may cause a transient increase in cytosolic Ca^2+^ concentration [10,18]. Figure 4A shows that Zeb1 upregulation by TGFβ was inhibited by either Ohmline (SK3 inhibitor) or the Ca^2+^ channel inhibitors GSK7975A and Synta66 (inhibitors of TRP and Orai channels, respectively). Ca^2+^ channel inhibitors had no effect on Zeb1 basal expression (Figure S5). As predicted, treatment with TGFβ increased the amplitude of SOCE. This effect was dramatically inhibited by LA and EPA, but not by PA (Figure 4B). A similar effect was obtained with Ohmline. This finding demonstrates that the TGFβ-induced SOCE is regulated by the SK3 channel.

**Figure 5:**
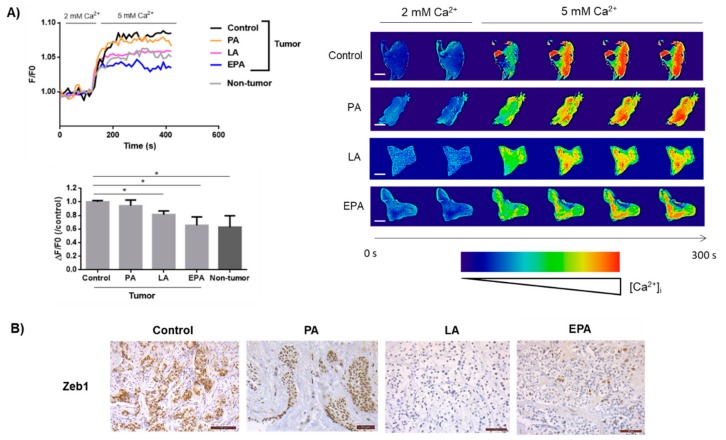
LA and EPA reduces Ca^2+^ entry in human PCa slices. (**A**) Human PCa slices (*N* = 9) and nontumor prostate slices (*N* = 4) were obtained from 11 patients, and were treated or not for 48 h by FA (60 µM). Slices were incubated with Rhod-2-AM in 2 mM CaCl_2_ PSS. After a stabilization period, CaCl_2_ was added to the bath to reach a final concentration of 5 mM Ca^2+^. Analyses were performed with ImageJ 1.52a analysis software. The Ca^2+^ fluorescence traces were expressed as F/F0 (F0: basal fluorescence signal obtained in 2mM Ca^2+^ ). Rate of real-time fluorescence images represented is 0.2 Hz. Scale bars = 2 mm. Diagrams represent the variations of Ca^2+^ signal expressed by ΔF/F0 (ΔF: maximal (5 mM Ca^2+^) − basal fluorescence (2 mM Ca^2+^)). Statistical differences are indicated: * *p* < 0.05 (Wilcoxon test). Results are expressed as mean ± SEM. (**B**) Organotypic cultures of human PCa slices with initial Zeb1 expression were obtained from 3 patients, and were treated with PA, LA and EPA (60 µM) for 48 h. When compared to control (almost 100% positive cells), Zeb1 expression remained identical after PA treatment, whereas both LA and EPA treatment led to decrease Zeb1 staining, with, respectively, no and 10% positive cells.

We next investigated whether these findings could also be observed in clinical samples. We previously developed a method to measure variations of intracellular Ca^2+^ dynamics in human prostate slices [19]. As observed in Figure 5, exposure of PCa slices to supraphysiological extracellular Ca^2+^ concentrations (from 2 to 5 mM) induced a robust and rapid increase of intracellular Ca^2+^ concentrations, which was followed by a plateau phase (curve control). As already observed in our previous study [19], Ca^2+^ entry was increased in tumor compared to nontumor prostate slices. Interestingly, PCa slices supplemented with LA and EPA exhibited a reduced responsiveness to extracellular Ca^2+^ variations to a level close to that observed in nontumor tissues. Accordingly, the increase of intracellular Ca^2+^ concentration was of a reduced amplitude in LA- and EPA-treated slices (*p* = 0.019 and 0.039, respectively; *N* = 9 for each condition) compared to vehicle alone-treated slices. In PA-treated slices, no significant change in Ca^2+^ entry was observed (*p* = 0.43, *N* = 9) (Figure 5). Figure 5B shows representative images of Zeb1 protein expression determined by immunohistochemistry in PCa slices treated with FA. In contrast to PA, LA and EPA supplementation strongly decreased Zeb1 staining.

## 3. Discussion

Expression levels of the key EMT transcription factor Zeb1 is increased with the stages of PCa progression, and is at its highest level in metastases. This factor has a major impact on patients’ survival [7]. We showed that Zeb1 is an important mediator of TGFβ-induced PCa cell migration. However, little is known regarding the key signal transduction pathways that serve as cytosolic connections between cell surface receptors and EMT nuclear transcription factors. Several studies have recently shown a transient increase in cytosolic Ca^2+^ concentrations mediated by major EMT inducers such as hypoxia, EGF [18], TGFβ [10,20,21], or androgens [22]. In the present study, we provide evidence that Zeb1 expression after EMT induction occurs following an increase in intracellular Ca^2+^ concentration.

Several studies have reported a modulation of Ca^2+^ channel expression as a consequence of EMT induction in cancer cells [9]. We report for the first time that expression of the SK3 channel is increased by EMT inducers such as TGFβ. We show that TGFβ-induced SK3 expression required the presence of Zeb1, which suggests that *KCNN3* (SK3 encoding gene) could be a target gene for Zeb1. Experiments on promoter activity demonstrated a transcriptional control of the SK3 channel gene by Zeb1. This finding is novel, as, up until now, little was known about the transcriptional regulation of the SK3 channel gene. Previous studies had shown a regulation by members of the Sp transcription factor family and estrogens [23]. Taken together and as summarized on Figure 4C, our results suggest a positive feedback loop between Zeb1 and the SK3 channel in a pathway that leads to increased Ca^2+^ entry that promotes PCa cell migration.

TGFβ can act as both activator and repressor of prostate cancer progression. TGFβ exerts a tumor suppressor role at the early stages. However, during tumor progression, TGFβ is involved in migration and invasion processes via the activation of EMT, which is involved in tumor progression and metastases [24,25]. In this study, we used TGFβ as an EMT inducer. As observed in the results section, TGFβ strongly induces the expression of EMT transcription factors (Zeb1, Snail, and Slug) and mesenchymal markers such as N-cadherin and metalloproteinase-9 (MMP-9) (Figure 2). These markers represent hallmark of the acquisition of an aggressive tumor phenotype. When Zeb1 is inactivated, we observed a decrease in mesenchymal marker and an increase of epithelial marker levels. These data show that Zeb1 is involved in the promoter effects of TGFβ. Among transcription factors, Snail is an important driver of EMT [5,26]. A strong induction of Snail by TGFβ was observed in PCa cells, but its expression was not regulated by LA and EPA. In a previous study, we have demonstrated that Zeb1 expression in human PCa tissues increased with disease progression from localized (pT2) to locally advanced (pT3) tumors and metastases [7]. In contrast, we did not observe any difference in Snail expression during tumor progression.

The present in vitro and ex vivo results suggest that LA and EPA exert their “anticancer” effects by reducing Ca^2+^ entry involved in Zeb1 regulation. This finding is crucial as *SK3* is a target gene of Zeb1. Indeed, we reported in a previous study a pivotal role for the SK3 channel in human cancer cell migration and bone metastases [14]. The mechanism by which LA and EPA inhibit SOCE remains to be determined. We hypothesize that these FA, through their incorporation into the plasma membrane, could disrupt lipid raft SK3- Ca^2+^ channels complexes, and this disruption may lead to decreased SK3-dependent Ca^2+^ entry and cell migration.

No clinical trial has reported the use of FA as adjuvant treatment in PCa. We have previously showed that, by analyzing FA profile of periprostatic adipose tissue (PPAT) obtained from PCa patients, the least aggressive tumors were those with the highest levels of LA and EPA [4]. FA concentrations used in the present study are consistent with those observed in PPAT. A clinical trial with n-3 FA dietary supplementation of metastatic breast cancer patients has reported that FA administration during chemotherapy was well-tolerated and safe, with an even slightly lower toxicity of the chemotherapy with respect to anemia and thrombopenia compared to control patients [27]. This observation is in agreement with our previous results obtained in experimental animal models in which we showed no significant difference in blood composition and animal weight in the group treated by a mix of n-3 FA [28]. Nutritional supplementation with LA of rodents does not have any toxic effect but have rather beneficial cardiovascular effects [29]. In human prospective observational studies, dietary LA intake is inversely associated with coronary heart disease risk in a dose-dependent manner [30]. Among n-6 FA, the effect of arachidonic acid (AA) is better documented, as AA and its related bioactive inflammatory mediators are rather considered as cancer promoters [31]. In the present study, we did not observe a rise in AA in membrane phospholipids from PCa cells after LA treatment. In addition, no difference in AA content was observed in human PPAT according to the LA level [4].

As animal models do not perfectly reproduce the clinical situations of PCa, we developed ex vivo tissue cultures of human PCa slices, which preserve both tumor microenvironment and cancer cell heterogeneity. We reported that this model reproduces the characteristics of human PCa, including its sensitivity to androgen deprivation and response to hypoxia and extracellular Ca^2+^ [19]. In our previous work, we compared intracellular Ca^2+^ concentrations between tumor and adjacent nontumor prostate slices and reported increased intracellular Ca^2+^ concentrations in malignant compared to benign tissues (about 20% increase). We also found greater intracellular Ca^2+^ concentrations in tissues sample obtained from aggressive PCa compared to indolent tumors. This finding suggested that Ca^2+^ entry may contribute to cancer progression [19]. Importantly, there is extensive evidence suggesting an important role for Ca^2+^ during tumorigenesis and cancer progression [32]. In the present study, we have demonstrated that LA and EPA can reduce Ca^2+^ entry not only in vitro, but also ex vivo, in human PCa slices. As these FA may reduce PCa aggressiveness through “normalization” of Ca^2+^ entry in cancer cells, they could therefore be used for therapeutic applications.

## 4. Materials and Methods

### 4.1. Human Tissue Slices

Prostatic tissue samples were obtained from 14 patients undergoing radical prostatectomy for PCa. The characteristics of patients and tissues are summarized in Table 1. Written informed consent was obtained from patients in accordance with the requirements of the medical ethic committee of our institute (ethic code DC-2014-2045). Immediately after surgery, 4–5 mm samples were dissected aseptically and cut with a vibratome into 6–10 slices per sample, as previously described [19]. Slices were incubated with DMEM medium supplemented with 10% fetal bovine serum (FBS), 1% penicillin–streptomycin, 1 nM dihydrotestosterone, and placed in a humidified incubator at 37 °C with 5% CO_2_. Slices were incubated in a humidified incubator at 37 °C with 5% CO_2_, and treated or not with fatty acids, LA, EPA, and PA (60 µM) for 48 h. Slices were used for either ex vivo culture and immunohistochemistry (3 patients) or for ex vivo culture and intracellular Ca^2+^ measurements (11 patients).

#### 4.1.1. Immunohistochemistry

Tissue slices or cells were fixed in 10% formalin, embedded in paraffin, and cut in serial 3 µm sections. One section was stained with hematoxylin-eosin-saffron (HES), and the other sections were deparaffinized, rehydrated, and heated in citrate buffer pH 6 for antigenic retrieval. After blocking for endogenous peroxidase with 3% hydrogen peroxide, Zeb1 primary antibody (Abnova, Taoyuan Taiwan, dilution 1/500) was incubated 1 h. Immunohistochemistry was then performed using the streptavidin–biotin–peroxydase method with diaminobenzidine (DAB) as the chromogen (Kit LSAB, Dakocytomation, Glostrup, Denmark). Slides were finally counterstained with haematoxylin. Positive staining was expressed as a percentage of total cancer cells.

#### 4.1.2. Ex Vivo Intracellular Ca^2+^ Measurements

Intracellular calcium variations were assessed with rhod-2 calcium-sensitive dye. Briefly, human prostate slices were incubated for 30 min with 5 µM of Rhod-2 AM (at room temperature). Then, slices were washed for 20 min with a physiological saline solution (PSS) before experiments. The PSS had the following composition (in mM); NaCl 140, MgCl_2_ 2, KCl 4, D-glucose 11.1, HEPES 10, and CaCl_2_ 2, adjusted to pH 7.4 with NaOH. The dye was excited at 545 ± 20 nm with LED illumination (CoolLED pE-300 white) and fluorescence was collected at 605 ± 40 nm with a sCMOS Zyla 4.2 PLUS camera and a Macro Zoom System Microscope MVX10. Image acquisition rate was 0.1 Hz, and exposure time was 600 ms. For measurement, human prostate samples were incubated in 2 mM CaCl_2_ PSS. After a stabilizing time (~100 s), CaCl_2_ was added to the bath to reach a final concentration of 5 mM Ca^2+^. Analyses were performed with ImageJ Software. The Ca^2+^ fluorescence signal was normalized by the initial fluorescence signal obtained in the 2 mM (CaCl) to correct differences of dye loading across the preparation, and then the variations of the Ca^2+^ signal was determined by calculating the difference between the basal, at 2 mM CaCl_2_, and the maximal Ca^2+^ fluorescence value after 5 mM CaCl_2_ application.

### 4.2. Cell Lines and Products

Human PCa DU145 (HTB-81) and PC3 (CRL-1435) cell lines was obtained from American Type Culture Collection (ATCC, Manassas, VA, USA) and was received on 2016/2018. This cell line has been tested and authenticated by DNA fingerprinting by the ATCC. After reception, cells were amplified in order to make a large reserve of cryopreserved cells. Every 3 months, a new cryopreserved bulb was thawed and used for this study. DU145 and PC3 cell lines were grown in RPMI medium (BE12-702F, Lonza, Levallois-Perret, France), supplemented with 5% FBS (CVFSVF0001, Eurobio, Les Ulis, France) and 1% (v/v) penicillin–streptomycin in a humidified incubator at 37 °C with 5% CO_2_. All experiments were performed with mycoplasma-free cells. DU145 and PC3 PCa cell lines have been chosen for their high migration capacity.

LA (L1876), EPA (17266), palmitic acid (PA) (P5177), and TGFβ1 (H8541) were from Sigma-Aldrich. GSK7975A (GLXC03243) and Synta66 (GLXC03244) were from GLXX Lab IMC. 1-Ohexadecyl-2-O-methyl-sn-glycero-3-lactose (Ohmline) was synthetized as previously described [33].

### 4.3. Cytosolic Ca^2+^ Measurements in Cell Lines

Cytosolic Ca^2+^ concentrations were studied using the ratiometric fluorescent dye Fura-2-AM (Molecular Probes) (1 h, 5 µM). For SOCE measurement, free-Ca^2+^ PSS (composition in Supplementary Methods) was added and, after a stabilizing time (around 100 s), cells were treated with thapsigargin (Tg 5 µM). After total endoplasmic reticulum Ca^2+^ depletion (~500 s), PSS with 2 mM CaCl_2_ (2Ca), was added. Fluorescence emission was measured at 510 nm with an excitation light at 340 and 380 nm. Analyses were performed using the SoftMax Pro Software. Results were expressed by the value at the peak 2Ca normalized by the value of the area of the Ca^2+^ Tg responses.

### 4.4. Reporter Gene Constructs and Luciferase Assay

*KCNN3* promoter sequence has been delineated following publication of Sun et al. that described limits of functional promoter (2540 bp) [34]. Transfection was performed with TransIT-2020 reagent (Mirus, Madison, WI, USA), 10ng of pRL-TK (Promega Madison, WI, USA), 50ng of pGL4.17-KCNN3, and 100ng of pCIneo-ZEB1 or pCIneo vector control (kindly provided by Marc Stemmler, Alexander University of Erlangen-Nürnberg, Erlangen, Germany). Cells were lysed 72 h after transfection with passive lysis buffer (Promega) and reporter assays were measured with Dual-Luciferase^®^ Reporter Assay System (Promega).

### 4.5. Migration Assays

Transwell assays were performed in cell culture inserts with 8µm pore size (353097, Falcon, France). Migrated cells to the bottom side were fixed, stained, and automatically counted. Wound healing was performed with a sterile 2-mm-wide tip on confluent cells. Phase-contrast images were performed on a Nikon microscope coupled to a Nikon camera (DS Qi2). The system included a cage incubator (Okolab, Pozzuoli, Italy) that controlled temperature and levels of CO_2_ and O_2_. Analyses were performed using the NIS Element AR software. Results were analyzed by measuring the area of the injured area to 0 h and 48 h after injury. Values were plotted as the percentage of wound closure.

### 4.6. siRNA Assays

Cells seeded at a density of 250,000 cells/well were incubated with a mix of siRNA and PepMute (SL100566, TebuBio, Le Perray-en-Yvelines, France) in medium for 6h. SiRNA used in this study have been validated in previous reports and are available in Supplementary Data [35,36]. Experiments were performed 48 h after transfection.

### 4.7. Quantitative Real-Time PCR.

Total RNAs from cultured cells were extracted using the Nucleopsin RNA kit (Macherey-Nagel, Hoerdt, France). RNA was reverse-transcribed with an RT kit (PrimeScriptTM RT Reagent, Perfect Real-time, Takara, Saint Germain en Laye, France). mRNA levels were quantified using LightCycler 480 (Roche Applied Science, Meylan, France). For each reaction, SYBR Green mix (RR420L, Takara) was mixed with specific primers (0.5 µM, Supplementary Methods) and cDNA at 50 ng/µL. Relative levels of mRNA were calculated according to the ΔΔCT method relative to the housekeeping gene HPRT and Cyclophilin A.

### 4.8. Western-Blot

Cells were lysed in RIPA buffer (50 mM Tris (pH 7.4), 1% NP-40, 150 mM NaCl, 1mM EDTA, 1 mM EGTA, 0.1% SDS, 0.5% sodium deoxycholate, and 10% glycerol) with phosphatase inhibitors (P2714, Sigma-Aldrich, St. Quentin Fallavier, France) and protease inhibitors (Thermo Scientific, Illkirch, France). Protein concentration was determined by BCA protein assay kit (23225, Thermo Scientific). Proteins were separated by denaturating SDS-PAGE and transferred to a polyvinylidene difluoride membrane (IPVH00010, Millipore, Molsheim, France). Primary antibodies were incubated overnight. The antibodies used were rabbit anti-Zeb1 (3396P; Ozyme, Saint Cyr l’Ecole, France), mouse anti-Ecadherin (5296S, Ozyme), and goat anti-β-adaptin (sc 6425, Santa Cruz, Dallas, TX, USA). Antibody binding was revealed with horseradish peroxidase conjugated anti-rabbit, anti-mouse, and anti-goat antibodies (Santa Cruz). The bands were detected with the Amersham ECL SelectTM Western Blotting Detection Reagent kit (RPN 2235, GE Healthcare, Sigma, St. Quentin Fallavier, France), visualized with a CDD camera (DNR Bio-imaging Systems MF-ChemiBis 3.2.) and quantified using Multi Gauge software.

### 4.9. Statistics

Analyses were made using the Mann–Whitney test and Kruskal–Wallis one-way ANOVA, followed by Dunn’s test and the nonparametric Wilcoxon (as indicated in figure legends).

## 5. Conclusions

The present study identified in PCa a signaling pathway leading to Zeb1 and SK3 induction through increased Ca^2+^ entry, and this pathway is associated with increased disease aggressiveness. Several steps of this process are strongly inhibited by LA and EPA supplementation. This observation underlines the therapeutic potential of these PUFAs as adjuvant for cancer treatments. 

## Figures and Tables

**Table 1:** Characteristics of patients and tissues.

Age (y), Median (Range)		63 (51–75)
PSA (ng/mL), Median (Range)		7.2 (4.5–35)
**pTNM stage**	**pT3**	n = 7
	**pT2**	n = 7
**ISUP score**	1	n = 1
	**2**	n = 4
	**3**	n = 5
	**4**	n = 2
	**5**	n = 2

## Supplementary Materials

The following are available online at https://www.mdpi.com/2072-6694/11/11/1814/s1. Table S1: Ca^2+-^free PSS composition; Table S2: siRNA sequences; Table S3: qPCR Primers; Figure S1: Effect of fatty acids on basal migration; Figure S2: Effect of fatty acids on basal Zeb1 expression; Figure S3: Effect of LA on the expression of TRPC1, STIM1, Orai1, and Orai3; Figure S4: Effect of fatty acids on basal SK3 expression; and Figure S5: Effect of Ca^2+^ channel inhibitors on Zeb1 basal expression.

## Fundings

This work was supported by grants from the Region Centre, Ligue Nationale contre le Cancer comités 37, 49, 56, and Institut National de la Santé et de la Recherche Médicale (Inserm), Canceropôle Grand Ouest. Sandy Figiel was the recipient of a fellowship from the Ministère de l’Enseignement Supérieur et de la Recherche.

## Abbreviations

EMT	Epithelial-to-mesenchymal transition
EPA	Eicosapentaenoic acid
FA	Fatty acid
LA	Linoleic acid
PA	Palmitic acid
AA	Arachidonic acid
PPAT	Periprostatic adipose tissue
PUFA	Polyunsaturated fatty acids
PCa	Prostate cancer
SOCE	Store-operated Ca^2+^ entry
